# A Robust and Highly Integrated Laser Doppler Velocimeter for High-Precision Velocity Measurement of Hot-Rolled Bars Under Thermal Radiation

**DOI:** 10.3390/s26134046

**Published:** 2026-06-25

**Authors:** Zimu Li, Lewen Zhang, Cheng Zuo, Jinhui Shi, Ming Fang, Yiren Wang, Wenbin Wu, Haibin Wu

**Affiliations:** 1 School of Electronic and Information Engineering, Hefei Institute of Technology, Hefei 238076, China; lizimu@hfit.edu.cn (Z.L.); czuo@hfit.edu.cn (C.Z.); shijinhui@hfit.edu.cn (J.S.); fangming@hfit.edu.cn (M.F.); whb62@163.com (H.W.); 2 School of Artificial Intelligence Engineering, Hefei Institute of Technology, Hefei 238076, China; wang.yiren@hfit.edu.cn (Y.W.); wwb0615@126.com (W.W.)

**Keywords:** laser Doppler velocimetry, hot-rolled bars, thermal radiation, spectral refinement, near-zero tension rolling

## Abstract

**Highlights:**

**What are the main findings?**

- A parallel-beam laser Doppler velocimetry system with narrow-band filtering is proposed to resist intense thermal radiation.
- Integration of continuous Fourier transform spectral refinement and centroid estimation yields a high accuracy of 0.005 m/s.

**What is the implication of the main findings?**

- Successful industrial deployment on a rolling mill achieves a near-zero tension rolling process.

**Abstract:**

Real-time, non-contact velocity measurement of hot-rolled bars is critical for metallurgical process control, but conventional laser Doppler velocimetry (LDV) systems often fail in these environments. The intense broadband thermal radiation from targets up to 1000 °C, coupled with severe surface depolarization, overwhelms weak scattered signals in high-speed (up to 40 m/s) rolling zones. To address this issue, we developed a fully integrated, thermal-radiation-resistant LDV sensing system. Hardware optimization was achieved by eliminating polarized-light transmission and adopting a parallel-beam design, which significantly enlarges the laser overlap area and increases detection depth. Furthermore, a 1550 nm laser (100 mW) was coaxially combined with a 10 nm narrow-band filter to isolate the thermal background and boost signal strength. A customized workflow utilizing continuous Fourier transform (CFT) spectral refinement and energy centroid estimation was implemented to precisely extract the true Doppler shift. Performance evaluations show the system achieves an excellent signal-to-noise ratio (SNR) of 29,532. Allan variance analysis confirms a stable detection sensitivity of 0.003 m/s (0.1 s integration time), a local short-to-medium-term optimal limit of 1.6 × 10^−4^ m/s, and a statistical accuracy of 0.005 m/s. Finally, the system was successfully deployed on an industrial rolling mill production line. It provided reliable velocity feedback for mill speed adjustment, achieving a near-zero-tension rolling process and fundamentally resolving workpiece dragging, squeezing, and steel pile-up.

## 1. Introduction

In the metallurgical industry, real-time and high-precision velocity measurement of hot-rolled bars is crucial. It is a key prerequisite for advanced process control, precise cutting, and product quality assurance [1,2,3]. Traditional contact-based sensors, such as encoders and measurement rollers, perform poorly in harsh industrial environments. They are highly susceptible to slippage, mechanical wear, and thermal damage. Consequently, they can induce surface tension in the hot-rolled bars. This severely degrades the final product quality [4,5,6]. Therefore, non-contact velocity sensing is urgently needed to upgrade intelligent metallurgical manufacturing.

Laser Doppler velocimetry (LDV) is a highly reliable non-contact sensing technology. It offers high accuracy and rapid dynamic response. Moreover, it operates independently of the target’s material or surface texture [7,8,9,10]. LDV measures instantaneous velocity directly. It relies on the passive Doppler shift in light scattered by moving surfaces or particles [11,12,13]. However, applying standard LDV systems directly to hot-rolled bar production lines remains challenging.

The primary obstacle is the intense thermal radiation from the target. Hot-rolled bars exit the furnace at approximately 1000 °C. At this temperature, they emit strong, broadband thermal radiation [14,15,16]. This thermal background enters the photodetector alongside the useful backscattered laser signal. In standard LDV optics, it acts as overwhelming broadband noise. Furthermore, the rolling process eventually reaches high-velocity zones (up to 40 m/s). Here, the bar diameter decreases sharply to roughly 20 mm. To address this issue, this device emits a parallel beam rather than one that converges at a single point. As a result, the overlap area of the laser is much larger than that of traditional two-point convergence systems, and the detection depth is significantly increased. The width of the laser beam emitted by the sensor is 10 mm. However, this also reduces the intensity of the received signal. Consequently, the effective backscattering cross-section drops rapidly. Extreme thermal noise thus combines with weak signal intensity. This completely drowns out the Doppler frequency peak. As a result, conventional LDV systems fail entirely. Robust measurement in these environments, therefore, requires two key improvements. First, the system must physically suppress high-temperature thermal interference. Second, it must maintain a high signal-to-noise ratio (SNR) for the velocity signal.

To address these challenges, this paper proposes a fully integrated LDV sensing system. It is specifically designed to resist thermal radiation while measuring high-temperature hot-rolled bars. The main contributions are as follows: (1) Optical path optimization: A 1550 nm narrow-linewidth laser serves as the light source. A narrow-band filter (10 nm bandwidth) is placed coaxially in the receiving optical path. This setup physically blocks most of the broad-spectrum thermal background. (2) Power and sensitivity enhancement: Narrowband filters have a peak transmittance of approximately 90%, so they do not significantly reduce light intensity. However, hot-rolled bars still emit strong radiation at a wavelength of 1550 nm, which cannot be completely filtered out. Therefore, the signal-to-noise ratio can only be improved by increasing the laser power. To compensate, the laser power was increased to 100 mW. This hardware upgrade significantly amplifies the amplitude and SNR of the Doppler signal. (3) Targeted signal processing: A custom signal processing workflow was implemented. It combines continuous Fourier transform (CFT) spectral refinement with an energy centroid estimation algorithm. This method refines the local spectrum near frequency peaks to accurately calculate the true Doppler shift. It ensures high-precision velocity inversion, even under residual noise disturbances.

Finally, the system components were integrated into a compact, industrial-grade housing. This includes the opto-mechanical structure, signal processing module, and display module. Field experiments were conducted on a working hot-rolled bar production line. The proposed LDV system successfully extracted high-quality velocity signals across varying velocity and temperature ranges. The optimized system achieved an excellent SNR of up to 29,532. This proves its exceptional robustness and reliability for industrial deployment.

## 2. Sensor Configuration

### 2.1. Measurement Principle

The laser Doppler velocimetry technique employs a dual-beam dual-scattering optical configuration. Two laser beams of equal intensity irradiate the same point on the object surface, forming an interference region [17,18]. Moving particles passing through the coherent zone scatter echo signals with Doppler frequency shifts [13,19]. These signals are received by a photodetector, which converts optical signals into electrical signals [20,21]. The object’s velocity is calculated based on the Doppler effect through frequency shift analysis.

A Gaussian laser beam exhibits a characteristic intensity distribution during spatial propagation, with maximum irradiance at the beam center and gradual attenuation toward the periphery. Collimated Gaussian light has a large depth of field and is effective at mitigating high levels of noise and vibration in the field. The laser output is split into two beams via a 50:50 fiber beam splitter, which converge at the same surface point to generate an interference region. When moving particles traverse this coherent zone at velocity *v*, the photodetector captures scattered echo signals from the particles and converts them into electrical signals. The photocurrent signal can be expressed as follows:

(1) $$i\left(t\right)=i_{d}\exp\left\{-{\left[\frac{2\sqrt{2}\left(t-t_{0}\right)}{\tau }\right]}^{2}\right\}+i_{a}\exp\left\{-{\left[\frac{2\sqrt{2}\left(t-t_{0}\right)}{\tau }\right]}^{2}\right\}\cos\left[2\pi f_{d}\left(t-t_{0}\right)\right]$$

where $i_{d}$ is the baseline signal amplitude, $i_{a}$ is the cosine signal amplitude, $\tau =D_{e}/\left(V\cos\theta \right)$ is the finite transit time of the particle through the control volume, $D_{e}$ denotes the beam waist diameter, $t_{0}$ represents the particle arrival time, $f_{d}$ corresponds to the Doppler frequency shift reflecting the particle velocity.

The objective of CFT-based spectral refinement is to augment the local density of spectral lines within a specific frequency region, thereby achieving enhanced spectral resolution. This improvement directly translates to higher accuracy in spectral estimation. By isolating a frequency segment centered at the spectral peak and symmetrically refining its adjacent regions, the refined spectrum can be mathematically expressed as follows:

(2) $$X\left(k\right)=\sum_{n=0}^{N-1}x\left(n\right)e^{-j\frac{2\pi f\left(k\right)n}{f_{s}}},\;k=1,2,3,\cdots L$$

In the formula, *k* represents each point of the refined frequency band, *N* represents the length of the signal, *x*(*n*) represents the original signal, *f*(*k*) represents the frequency of the corresponding point, *L* = *f*_1_/*f*_0_ represents the number of points in the refined spectral segment, *f*_1_ represents the refined spectral width, *f*_0_ represents the spectral resolution after refinement; *f_s_* represents the sampling frequency.

The energy centroid estimation method calculates the frequency value corresponding to the spectral centroid, which serves as an approximation of the true spectral peak position. After spectral refinement, a coarse estimation of the spectral peak is performed. A threshold (For instance, 5% of the peak amplitude) is then applied to isolate the spectral region for centroid estimation by selecting components exceeding this threshold. The centroid frequency is subsequently computed, and the corresponding Doppler frequency shift *f_d_* at the centroid can be expressed as follows:

(3) $$f_{d}=\frac{\sum_{i=x-N_{1}}^{x+N_{2}}\left(f_{i}A_{i}\right)}{\sum_{i=x-N_{1}}^{x+N_{2}}A_{i}}$$

where *f_i_* represents the frequency value at the *i*-th point, *x* is the label corresponding to the estimated spectral peak, $i\in \left[x-N_{1},X+N_{2}\right]$ is the estimated range taken, and *A_i_* is the spectral amplitude. When the range of the spectrum to be refined is very large, excessive refinement will increase the computational load. After a certain amount of spectrum refinement, frequency estimation can be carried out using centroid estimation, which can also achieve the same accuracy. Finally, the velocity of the object’s motion is inverted using the following [22]:

(4) $$V=\frac{\lambda f_{d}}{2\sin\theta }$$

where θ is half of the angle between the two laser beams, *λ* represents the wavelength of the laser.

### 2.2. Design of Velocimetry System

**Figure 1:**
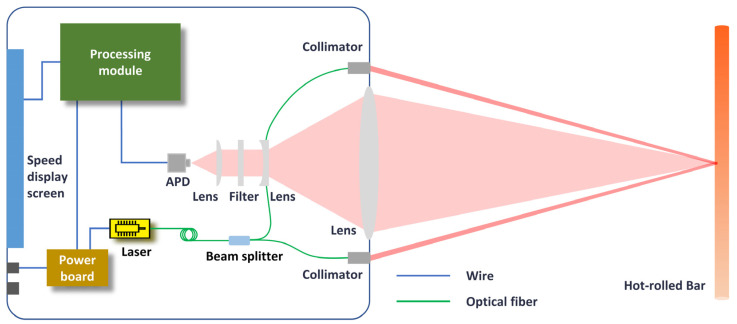
Schematic of the laser Doppler velocimetry system.

The developed laser Doppler velocimetry system and its opto-machine structure schematic diagram are depicted in Figure 1. The detailed components include a power supply board, a narrow linewidth laser (1550 nm, <50 kHz linewidth, output power is 100 mW), a beam splitter, a collimator, a lens set, a narrow band filter, an avalanche photodetector (APD), a signal processing module, and a liquid crystal display. When the system is powered on, the power board supplies power to the laser and causes it to emit a 1550 nm frequency-stabilized laser. The laser is divided into two paths using a 50:50 fiber beam splitter. After passing through the collimator and adjusting the angle reasonably, the two laser beams form a cross point directly in front of the center of the objective lens. A suitable location is chosen to place the LDV system, while making sure that the crossing point of the lasers falls on the horizontal surface of the hot-rolled bar. The scattered light from the hot-rolled bar surface then returns in the horizontal direction. It is received through the lens set and focused on the avalanche photodetector. It is worth mentioning that we have incorporated a narrow-band filter in the lens set. The purpose is to minimize the interference of the thermal radiation from the hot-rolled bars on the sensing system. Subsequently, the photoelectric signal goes to the signal processing module for computational processing and velocity inversion. Ultimately, the measured velocity data is displayed using a screen display.

**Figure 2:**
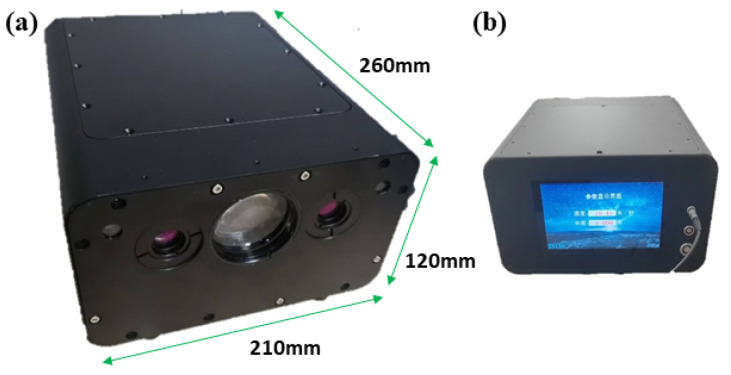
Physical drawing of the laser Doppler velocimetry system. (**a**) the front view; (**b**) the rear view.

The physical object of the LDV measurement system is shown in Figure 2. Figure 2a shows the front view, where the lens on the panel serves as the laser’s transmission and reception window. Figure 2b shows the rear view, where the window on the panel is a screen that displays the velocity. In order to facilitate the installation and use in the actual project, it is necessary to reduce the size of the system appropriately. Therefore, first of all, through a reasonable circuit board optimization design. The integration of the system’s optoelectronic signal processing circuit board is realized. Secondly, the collimated optical path with an adjustable angle is designed. Fiber optics is used to connect the laser and the collimator. This minimizes the space occupation ratio of the emitting optical path. Finally, the overall shell of the system with rounded edges was designed. Its specific shell dimensions are 260 mm × 210 mm × 120 mm. An oxidized, blackened metal housing is used to protect the internal components. Thus, the overall system package is integrated.

### 2.3. Signal Processing

**Figure 3:**
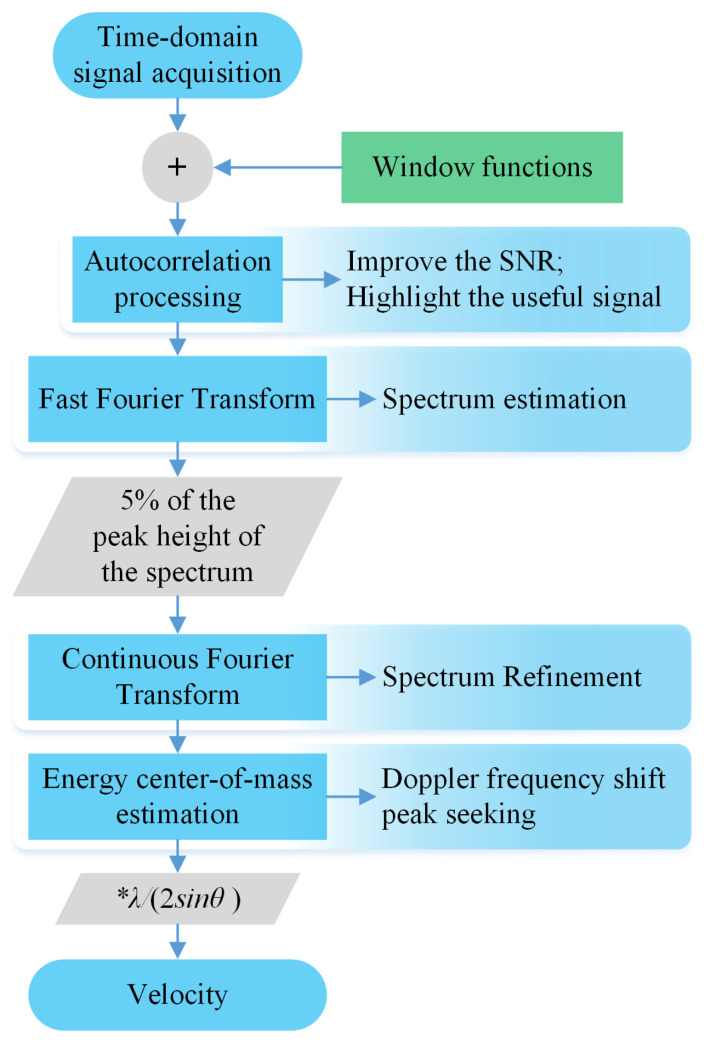
Flow chart of signal processing.

The signal processing flow is shown in Figure 3. First, the board acquires the time domain signal output from the APD. After being superimposed with the window function, the interception data is completed. And the data autocorrelation processing of the previous step. This can reduce the noise and improve the SNR. The purpose of highlighting useful signals is achieved. The fast Fourier transform (FFT) is applied to the useful signal to realize spectrum estimation. In order to reduce the amount of data computation. Only 5% of the peak height of the spectrum is selected for subsequent processing. Refining the spectrum near the peak can better find the Doppler shift position. This is performed by processing the above data with the help of the Continuous Fourier Transform. Subsequently, the energy center-of-mass estimation method is utilized to achieve the peak seeking of the Doppler frequency shift, after deriving the Doppler frequency shift *f_d_*. Ultimately, it is multiplied by the coefficient λ/(2sinθ), and the velocity value can be calculated and displayed on the screen.

## 3. LDV Sensing System Performance

### 3.1. Sensing System Improvement and Optimization

**Figure 4:**
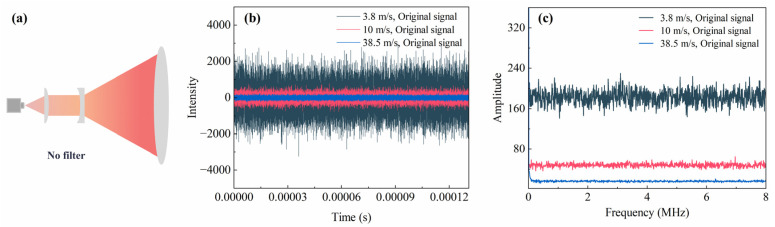
The originally designed LDV system. (**a**) optical path structure, (**b**) time-domain signal, and (**c**) frequency-domain signal.

The initially designed LDV system is shown in Figure 4. During the experiment, the prototype used a polarized-light transmission-and-reception setup. A narrow-linewidth laser (1550 nm, <50 kHz linewidth, output power is 30 mW) beam is first split and then collimated before being directed onto the target object. Next, using the three lenses shown in Figure 4a, the signal light scattered from the target object is focused onto the photosensitive surface of the APD. The sensors were initially tested in the laboratory. A rotating turntable driven by a controllable permanent-magnet brushless motor was used as the velocity measurement target. The signals were observed at different velocities. When the rotational velocity of the turntable was adjusted, the frequency peaks in the frequency domain correspondingly changed. The integrated LDV sensor system was then deployed at an industrial site. Experiments were conducted to collect velocity signals from high-temperature hot-rolled bars during the metallurgical process. Since the diameter of the hot-rolled bars varies depending on their position in the rolling process, their forward velocities also differ. Therefore, signals were collected at three positions with different velocities (3.8 m/s, 10 m/s, and 38.5 m/s). The collected time-domain signals are shown in Figure 4b. A comparison of signal strengths at the three velocities revealed that the signal strength for hot-rolled bars was strong in the low-velocity zone but weak in the high-velocity zone. The frequency domain information obtained via the FFT is shown in Figure 4c. The figure also does not show any frequency peaks corresponding to the velocity values. Based on signal analysis principles, this may be due to the background signal being too strong, causing the velocity signal to be overwhelmed by the background noise. Taking the on-site operating conditions into careful consideration, since the bar temperature upon exiting the furnace reaches approximately 1000 °C, thermal radiation conditions are present. Furthermore, the low-velocity zone represents the initial stage of rolling, where the bar diameter is relatively large (approximately 350 mm, and the corresponding velocity is 3.8 m/s). For the LDV sensing system, this results in a larger radiation-receiving area, leading to a higher signal intensity in the time domain. Similarly, as the rolling process proceeds, the bar enters the subsequent high-velocity zones at the 10 m/s and 38.5 m/s marks. At these points, the diameter of the hot-rolled bar decreases to approximately 200 mm and 20 mm, respectively. The temperature also drops to approximately 200 °C and 90 °C, respectively. The bar’s ability to radiate heat also diminishes. In Figure 4b, the signal intensities at 10 m/s and 38.5 m/s are lower than that at 3.8 m/s. Signal intensity shows an inverse relationship with velocity. This is because higher velocities correspond to lower temperatures at those locations, resulting in lower thermal radiation intensity. However, spectral analysis reveals that the useful velocity signal remains buried in the background noise. Therefore, the issue of signal interference caused by thermal radiation background noise must be addressed urgently.

**Figure 5:**
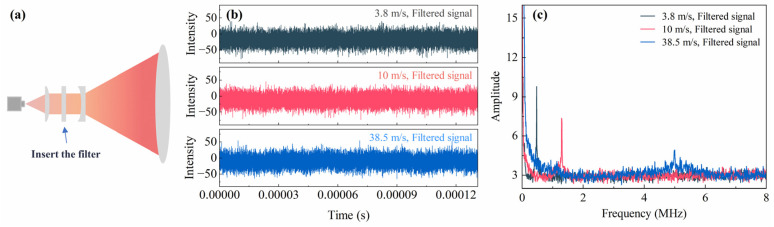
An improved LDV system with an inserted filter. (**a**) Optical path structure, (**b**) time-domain signal, and (**c**) frequency-domain signal.

To address the aforementioned issues and challenges, the receiving optical path of the LDV sensing system was optimized and improved, as shown in Figure 5a. The receiving optical path system employs a coaxial configuration. A narrow-band filter (center wavelength 1550 nm, bandwidth 10 nm) is placed coaxially within the parallel beam to the right of the focusing lens. To verify the feasibility of this approach, data acquisition experiments were also conducted at 3.8 m/s, 10 m/s, and 38.5 m/s. The resulting time-domain data are shown in Figure 5b. A comparison with Figure 4b reveals that the signal intensity values of the improved sensing system are lower. However, the signal intensities are essentially consistent across the three velocities. This indicates that this approach is effective in filtering out thermal radiation background noise. Subsequently, an FFT was applied to the time-domain signals, yielding the spectral results shown in Figure 5c. Each velocity corresponds to a frequency peak, and the results are consistent with Equation (4).

Narrowband filters typically have a transmittance of 90%. This results in a 10% loss in received light intensity. The detection scheme of this LDV sensing system relies on backscattered light. We define the spectral SNR in the frequency domain as *SNR* = *A_peak_*/*σ_noise_*. Where *A_peak_* is the peak amplitude of the Doppler shift, and *σ_noise_* (1*σ*) is the standard deviation of the noise baseline. As shown in Figure 5c, although a spectral signal is obtained when the laser power is 30 mW, its SNR is approximately 14.8. As the measurement velocity increases, the frequency peak becomes lower and lower, and the SNR correspondingly decreases. Consequently, when the velocity reaches a certain threshold, the frequency peak becomes buried in the noise. However, hot-rolled bars still emit strong radiation at a wavelength of 1550 nm, which cannot be completely filtered out. Therefore, the signal-to-noise ratio can only be improved by increasing the laser power. To address these issues of light intensity loss and low SNR, and after careful consideration of the opto-mechanical structure, this system will replace the original light source with a new laser. The laser power will be increased from 30 mW to 100 mW.

**Figure 6:**
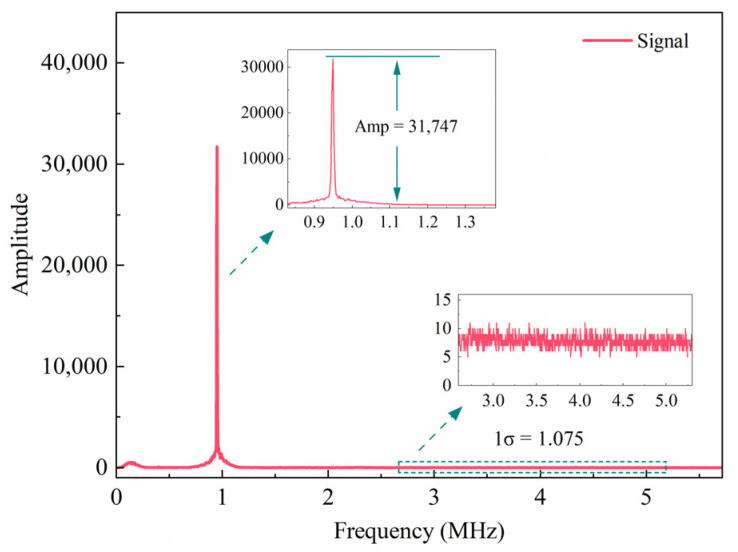
Measured hot-rolled bar motion signals.

At 1550 nm, surface evanescent fields and localized plasmonic interactions occur at the rough, oxidized boundary. The evanescent waves decay rapidly and do not reach our far-field receiver, while localized multi-scattering randomizes the polarization state. This depolarization physically justifies our polarized-free detection scheme. Notably, these near-field effects only influence signal intensity and polarization, leaving the macroscopic Doppler shift frequency completely unaffected. Due to the severe depolarization caused by the rough metal surface, no signal could be received. The improved sensing system eliminated the polarized-light transmission and reception scheme. In principle, interference from optical and thermal radiation backgrounds cannot be completely eliminated. According to Planck’s Law, the spectral radiance of the steel surface at 1000 °C at *λ* = 1.55 μm is $1.51\times 10^{3}\;W\cdot m^{-2}\cdot {\mu m}^{-1}$. Figure 6 shows a spectral amplitude signal collected at an industrial site. The specific velocity corresponding to Figure 6 is 7.24 m/s; the temperature of the rod here is approximately 800 °C. The flanking data exhibits noticeable fluctuations after amplification, indicating the presence of residual amplitude modulation. Through the analysis of the above data, the 1σ of the flanking data is about 1.075, and the amplitude of the frequency peak signal is 31,747. The calculated SNR is 29,532. After increasing the laser power to 100 mW, the SNR of the LDV system has improved significantly. The SNR was approximately 2000 times that shown in Figure 5c. This is more than sufficient for use in industrial environments with thermal radiation backgrounds. The capture card has a bandwidth of 75 MHz. To ensure detection accuracy and avoid amplitude attenuation and phase distortion near the cutoff frequency, we conservatively limit the maximum detection range to less than half of the bandwidth, i.e., approximately 30 MHz. When the maximum detection frequency is 30 MHz, the system’s maximum measurable velocity is calculated to be 234 m/s based on the Doppler relationship in Equation (4). This is still nearly six times the maximum on-site rolling speed (40 m/s), demonstrating that this system has an extremely generous and safe measurement range.

### 3.2. Analysis of Allan Variance and Measurement Precision

**Figure 7:**
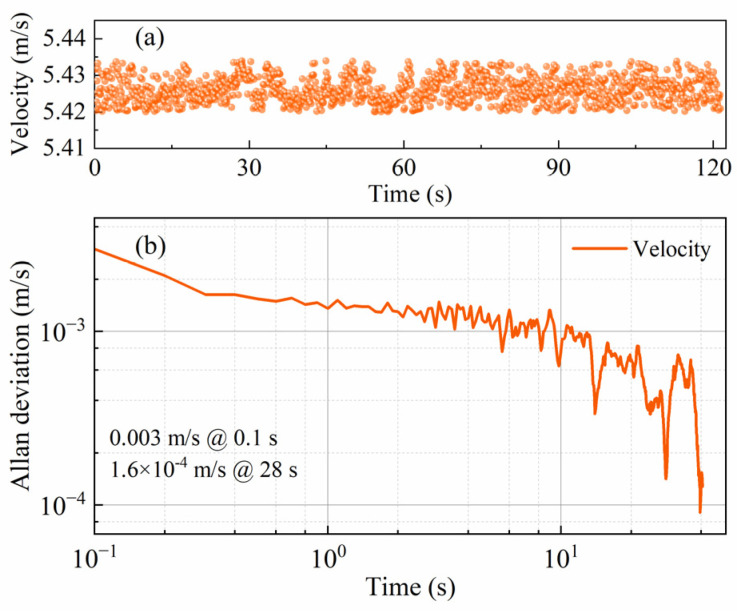
(**a**) Continuous collection of velocity values, (**b**) Allan deviation of velocity.

To investigate the noise sources, detection limits, and stability of the laser Doppler velocimetry system, a permanent magnet motor was set to rotate at a constant speed of approximately 5.425 m/s. Time-series velocity data were collected over a 121 s period at a sampling rate of 10 Hz, as shown in Figure 7a. A value is collected every 0.1 s. It can be seen that the measured velocity values exhibit significant stability, fluctuating slightly around a constant value throughout the measurement period. Figure 7b shows the results of the Allen variance analysis of the measurement data. The results indicate that, with an averaging time of 0.1 s, the detection sensitivity of the LDV system is 0.003 m/s. Due to the influence of random noise, the Allen variance of the LDV-measured velocity gradually decreases over time. The system reached an empirical minimum Allan deviation of 1.6 × 10^−4^ m/s at an averaging time of 28 s within the 121 s measurement window. Due to the limited number of independent samples at larger averaging times (≥28 s), this minimum should be interpreted as a local short-to-medium-term optimal limit carrying an estimated ±39.0% statistical uncertainty, rather than the absolute long-term stability asymptotic floor of the sensor.

**Figure 8:**
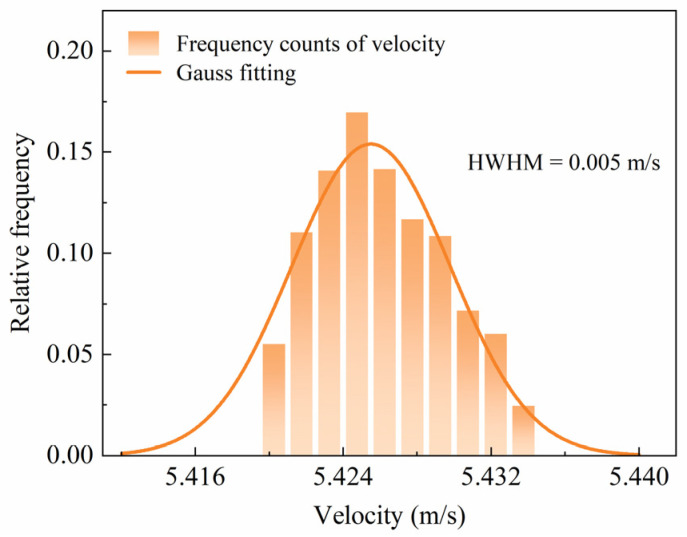
Statistical histogram and Gaussian distribution of velocity.

A statistical analysis was performed on the measured velocities shown in Figure 7a, and a histogram and a Gaussian-fitted curve were plotted, as shown in Figure 8. The distinct Gaussian distribution curve indicates that the measurement noise also follows a Gaussian distribution. The distribution of results fits the Gaussian curve well, with an R^2^ of 0.92. The half-width at half-maximum (HWHM) is 0.005 m/s. This indicates that the measurement accuracy of the LDV sensor is 0.005 m/s. These analytical results demonstrate that the sensor using this approach exhibits good stability and accuracy in the quantitative detection of bar velocities under thermal radiation backgrounds. Our system’s errors are quantitatively evaluated by isolating random and systematic components. For random error (precision), continuous measurement at a constant velocity of 5.425 m/s shows that the noise fits a Gaussian distribution (R^2^ = 0.92) with a HWHM of 0.005 m/s, representing an exceptionally low relative random error of only 0.092%. For systematic error (accuracy), primarily driven by angular alignment drift and wavelength instability, the mechanically locked opto-mechanical housing restricts physical angular drift to <0.05%, while the CFT + ECE algorithm achieves sub-bin spectral resolution to reduce discrete algorithmic systematic errors (the picket-fence effect) to near zero.

### 3.3. Measurement Results and Discussion of Hot-Rolled Bars

A rolling mill is a piece of equipment used to perform the metal rolling process. Its fundamental operation involves applying radial pressure to the metal using rotating rollers, causing indirect compressive deformation within the roll gap. This reduces the cross-sectional area and increases the length of the material. As core equipment in the steel and non-ferrous metal processing industries, rolling mills are widely used in fields such as construction, automotive, and aerospace. Their performance directly determines the precision and quality of the final product. A flying shear is a mechanical device that performs transverse shearing on a workpiece while it is in motion. It is widely used in continuous production lines for metallurgy, steel rolling, and wire manufacturing. The working principle of a flying shear involves driving the shear blades via a crank-connecting rod mechanism, rollers, or a combination of these mechanisms, enabling them to move in sync with the workpiece and perform the shearing operation. The key lies in matching the velocity of the shear blades with that of the workpiece. In traditional flying shears, the cutting velocity adjustment relies on the velocity of the line rollers. However, if hot-rolled bars slip on the line rollers, the shear velocity cannot be matched with the workpiece velocity. When hot-rolled bars are rolled through multiple rolling mills, if the velocities of the individual mills cannot be consistently matched, this can easily lead to the workpiece being dragged or squeezed, resulting in surface tension on the hot-rolled bars. In severe cases, this may even trigger steel pile-up accidents.

**Figure 9:**
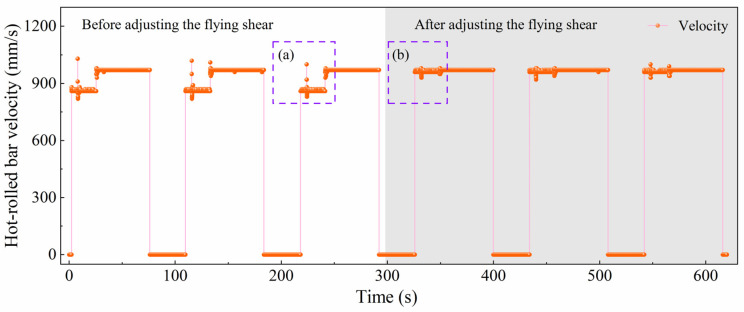
Changes in the velocity of hot-rolled bars before and after adjusting the velocities of the rolling mill and flying shear. (**a**) Hot-rolled bar velocity prior to adjusting the rolling mill and flying shear; (**b**) Hot-rolled bar velocity after adjustments to the rolling mill and flying shear.

Figure 9 shows the changes in the velocity of hot-rolled bars before and after adjusting the flying shear and rolling mill speeds. In actual production, due to the limited length of individual workpieces, the measurement point was placed midway between the two rolling mills. As shown in Figure 9, the velocity value reads zero when no workpiece is passing through the measurement point. The data within the box in Figure 9a reflects the production process prior to adjusting the rolling mill and flying shear velocity: (1) After passing through the previous rolling mill, the workpiece proceeds normally at a velocity of 860 mm/s. (2) When the workpiece is sheared by the flying shear blades, the velocity value undergoes a brief spike (approximately 1010 mm/s) due to the compressive and tensile forces during the shearing instant, after which it immediately returns to the normal velocity (860 mm/s). (3) The workpiece then enters the next rolling mill. Because this mill operates at a higher velocity, it drives the entire workpiece to travel at 970 mm/s. The mismatch in rolling mill velocities generates a certain amount of radial drag force. This situation can easily induce a certain degree of surface tension on the workpiece.

However, based on the measurement data from the LDV sensor system. After adjusting the velocities of the rolling mill and flying shear to match the actual workpiece running speed, the velocity changes in the workpiece are shown in the inset of Figure 9b. Throughout the early, middle, and late stages of the rolling process, the running speed of the entire workpiece remains relatively stable (approximately 970 mm/s). The magnitude of velocity jumps during flying shear cutting was also significantly reduced. This achieved a rolling process with near-zero tension. There is no risk of billet accumulation. Product quality has been improved, the working pressure on the rolling mill has been reduced, and the service life of the rolling mill has been extended. This is precisely the significance of measuring the velocity of hot-rolled bars.

## 4. Conclusions

To address the challenges of velocity measurement posed by intense thermal radiation from hot-rolled bars, this paper presents a fully integrated and highly stable LDV sensing system. Through systematic analysis, the failure mechanisms of traditional LDV systems in high-temperature and high-speed rolling zones were identified. To overcome these issues, a comprehensive opto-mechanical and algorithmic co-design was implemented. Optically, the polarized light transmission and reception scheme was eliminated to avoid signal loss caused by severe surface depolarization. A parallel beam design was adopted to enlarge the laser overlap area and significantly increase the detection depth. Furthermore, a 1550 nm laser (100 mW) was coaxially combined with a 10 nm narrow-band filter. This architecture effectively isolates the broadband thermal background and boosts the useful signal. Algorithmically, the system integrates continuous Fourier transform spectral refinement with an energy centroid estimation method. This ensures the precise extraction of the true Doppler shift, even under residual noise disturbances. Performance evaluations confirm that the optimized LDV achieves an excellent SNR of 29,532. Allan variance analysis demonstrates a stable detection sensitivity of 0.003 m/s at a 0.1 s integration time. The local short-to-medium-term optimal limit reaches 1.6 × 10^−4^ m/s at 28 s. Statistical analysis further verifies a high measurement accuracy of 0.005 m/s. In practical industrial applications, the sensing system was successfully deployed on a production line involving rolling mills and flying shears. By providing reliable velocity feedback, the system enabled precise speed matching between the shear blades and the workpiece. It maintained a stable running speed of approximately 970 mm/s throughout the rolling stages and significantly reduced velocity jumps during cutting. This capability achieves a near-zero-tension rolling process and eliminates the risk of billet accumulation. Consequently, it improves product quality, reduces working pressure on the equipment, and extends the service life of the rolling mill. The proposed thermal-radiation-resistant LDV system demonstrates remarkable robustness and immense reliability for intelligent metallurgical manufacturing.

## Data Availability

Data is contained within the article. The original contributions presented in this study are included in the article. Further inquiries can be directed to the corresponding author.
